# Improving Learning Performance with Happiness by Interactive Scenarios

**DOI:** 10.1155/2014/807347

**Published:** 2014-01-16

**Authors:** Chi-Hung Chuang, Ying-Nong Chen, Luo-Wei Tsai, Chun-Chieh Lee, Hsin-Chun Tsai

**Affiliations:** ^1^Department of Applied Informatics, Fo Guang University, Ilan, Taiwan; ^2^Department of Information Management, National United University, No. 1, Lienda, Miaoli 36003, Taiwan; ^3^Department of Information and Communications Research Lab, Industrial Technology Research Institute, Hsinchu, Taiwan; ^4^Department of Computer Science and Information Engineering, National Central University, Taoyuan, Taiwan; ^5^Department of Electrical Engineering, National Cheng Kung University, Tainan, Taiwan

## Abstract

Recently, digital learning has attracted a lot of researchers to improve the problems of learning carelessness, low learning ability, lack of concentration, and difficulties in comprehending the logic of math. In this study, a digital learning system based on Kinect somatosensory system is proposed to make children and teenagers happily learn in the course of the games and improve the learning performance. We propose two interactive geometry and puzzle games. The proposed somatosensory games can make learners feel curious and raise their motivation to find solutions for boring problems via abundant physical expressions and interactive operations. The players are asked to select particular operation by gestures and physical expressions within a certain time. By doing so, the learners can feel the fun of game playing and train their logic ability before they are aware. Experimental results demonstrate that the proposed somatosensory system can effectively improve the students' learning performance.

## 1. Introduction

Nowadays, the education background becomes increasingly important. Therefore, more and more parents have paid attention to early childhood education because they want their children to win at the starting line. However, some problems usually occur in the ordinary course of the children's educational process, such as learning carelessness, low learning ability, lack of concentration, and difficulties in comprehending the logic of math. Some children have even suffered from learning disorders, which their parents are unaware of. The phenomenon of children with learning disabilities, also known as learning difficulties, refers to children's intelligence in the normal range, but they have difficulty in learning. The common examples are mathematics disorder, dyslexia, writing disorders, attention-deficit/hyperactivity disorder, and so forth, which are all learning disabilities. The crucial point of guiding children to successful learning is to arise their interest. According to the market research of NPD, among 2 to 17 years old children and teenager population in America, 82% are video game players, amount to about 557 million people (2009), which demonstrates that video games are very popular leisure activities in the children and teenagers. Therefore, embedding the learning process in the video games playing would be a solution for happy learning. Hogle [1] proposed some advantages of learning by video games playing.

1. Trigger intrinsic motivation and increase interest: the nature of curiosity, expectation, control, interaction, and fantasy storyline in the video games could improve the learners' interest in learning and intrinsic motivation, and the learners would be able to keep trying in the face of difficult challenges for obtaining sense of achievements.
2. Memory reserving: compared with the traditional learning methods, learning by video games playing could achieve higher effect of memory reserving.
3. Practice and feedback: many learning by video games playing software provide learners chances of repeating practice and immediate feedbacks of errors, which make learners assess their learning performance and improve the achievement of learning objectives.
4. Proving high-level thinking: learning by video games playing is the best way of learning, since the design of video games meets with the human cognitive structure, which makes learners find solutions and obtain knowledge via repeatedly solving problems, making decisions, and integrating what they learn. Then, the teaching content could be constantly implanted into learners' memories.

Somatosensory system is an arising interactive video game and multimedia technology. The users can receive abundant feedbacks in the operation since its direct responding. Therefore, it would be a good choice to use experience system as a learning platform so that the students can learn happily and enhance their attention and interest in learning. Motivated from the above, the Microsoft Kinect is used to develop a learning somatosensory system in this study. The Kinect can extract color image, 3D depth image, and voice. In the proposed system, the 3D depth image is used to detect users' actions. The Kinect uses three steps for object detection and tracking. First, it uses Light Coding method to extract 3D depth image [2]. Then, the color image and depth image are combined to find out the human skeleton and joints [3]. Finally, the regression method is applied to improve the consistency between human posture and skeleton [4]. In addition, it can detect at most six people and recognize the actions of the two simultaneously. Twenty joints of skeleton are extracted for each detected and tracked people, including their body, limbs, and fingers for interactive somatosensory operation. Based on the extracted and tracked skeletons, pose estimation, [5–8], action recognition [9–12], image segmentation [13–15], body pose reconstruction [16–19], and building rich 3D maps of environments [20, 21] could be achieved. Although the Kinect defines and extracts many human joints, however, the details of the palm are insufficient. Therefore, the gestures cannot be detected and recognized in the original Kinect system. To solve this problem, in our proposed system, the palm joint is set as region of interest (ROI) via the Kinect skeleton tracking system and then extracted for image processing and gesture recognition via OPENNI environment.

In this study, we propose a learning somatosensory system based on Kinect to make children and teenagers happily learn in the course of the games and improve the learning performance. We propose two interactive geometry and puzzle games. The proposed geometry game can make learners feel curious and raise their motivation to find solutions for boring geometry problems via abundant physical expressions and interactive operations. The players are asked to select particular operation by gestures and physical expressions within a certain time. By doing so, the learners can feel the fun of game playing and train their logic ability happily. The proposed puzzles game can train the learners' concentration ability and logical thinking via abundant physical expressions. For example, when the learners are playing the puzzle games, they would practice their cognition ability to identify and group the shape and color of puzzles. In addition, the learners would practice their physical skills moving puzzles to correct positions via physical expressions and gestures.

The rest of this paper is organized as follows. In Section 2, the basis of Kinect system and digital learning will be briefly reviewed. Then, the proposed learning based on Kinect is presented in Section 3. In Section 4, experimental results are illustrated to demonstrate the soundness and effectiveness of the proposed digital learning method. Finally, conclusions are given in Section 5.

## 2. Related Works

### 2.1. The Application of Kinect

**Figure 1:**
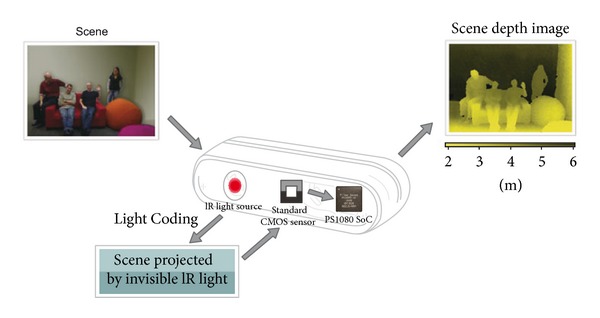
The procedure of object detection of Light Coding method (PrimeSense).

As mentioned above, the Kinect uses Light Coding method for object detection. This method is based on the Laser Speckle theory, which is a random reflecting speckle pattern produced when the laser light projects on some object. Since the speckle pattern would never be the same in any position, the monitored space is all marked to detect object's position. In practice, the Kinect uses infrared laser light projector and sensor to analyze the shift of laser speckle pattern by projecting from one position and observing from another to construct the depth map as Figure 1.

**Figure 2:**
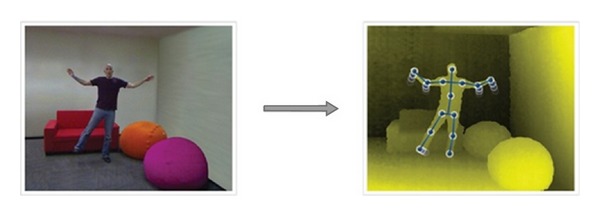
The skeleton of human body and tracking (PrimeSense).

After object detection and 3D depth map construction, the skeleton tracking system on the Kinect is applied to extract twenty joints of human body and limbs. Since the joints of human body and limbs are extracted, the relationships of the joints are used as features for action recognition in Microsoft somatosensory games. Based on the skeleton tracking system, the human can be tracked in real time as Figure 2 and other applications can also be achieved.

### 2.2. The Digital Learning

**Figure 3:**
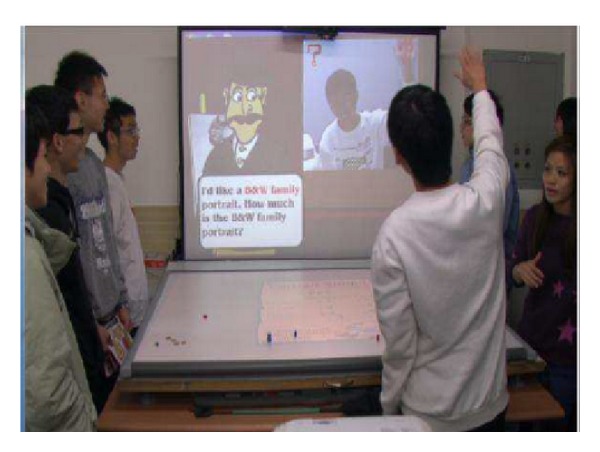
L-shape plate form [24].

**Figure 4:**
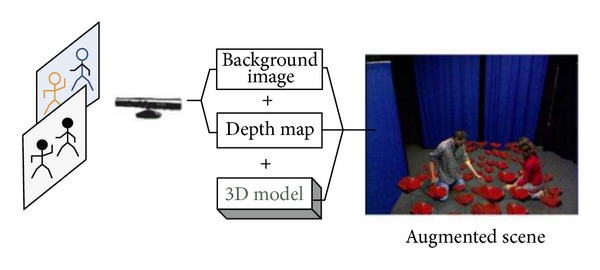
Integration of augmented reality and Kinect [25].

Recently, to enhance learning through games has attracted a lot of researchers. Sarmanho et al. [22] proposed a Kinect-based game to help students of dyslexia and dysgraphia. Kenneth et al. [23] integrated the drawing games and somatosensory system, so the students could paint via moving their limbs. Since the somatosensory system is a new user interface making the interactions between computers and users are improved. Therefore, Lien et al. [24] designed an L-shape platform for learning based on interaction with computers. As Figure 3 shows, students can easily and happily learn by moving their limbs and interacting with computers. Casas et al. [25] integrated augmented reality technology with Kinect to create an environment in which users can interact with virtual objects with limbs (as Figure 4 illustrates). In addition, in order to improve the willingness of students in learning, Smorkalov et al. [26] integrated virtual reality technology with Kinect to immerse users in the virtual world and operate the virtual characters with limbs. Tuveri et al. [27] proposed a method using gestures and positions of palms to control planetarium software, improving the effectiveness of learning.

Li [28] proposed a Protractor scheme which was a template-based and single-stroke gesture identifier that used a new closed-form method to compute the similarity between gestures. Kratz and Rohs [29] designed a $3 gesture recognition system using 3D acceleration sensors. The scheme was proposed to be implemented immediately in prototyping environments and does not need any special equipment or environments. Only simple trigonometric and geometric calculations were necessary. Kratz and Rohs [30] also proposed a lightweight classifier for motion-based 3D gestures solving the difficulty of searching the optimal rotation between an input gesture and a template gesture. Hong et al. [31] designed a state based method for gesture learning and recognition. It uses spatial clustering and temporal alignment, and gestures are defined as sequence of states in spatial-temporal space. Wobbrock et al. [32] proposed a $1 recognizer system for gestures recognition. The system was easy to be implemented. Although the scheme was simple, it can provide rotation, scale, and position invariance. It also needed no sophisticated mathematical operations but competed with methods using dynamic programming and statistical recognition.

## 3. The Proposed Somatosensory Learning System

### 3.1. The Details of Kinect System

**Figure 5:**
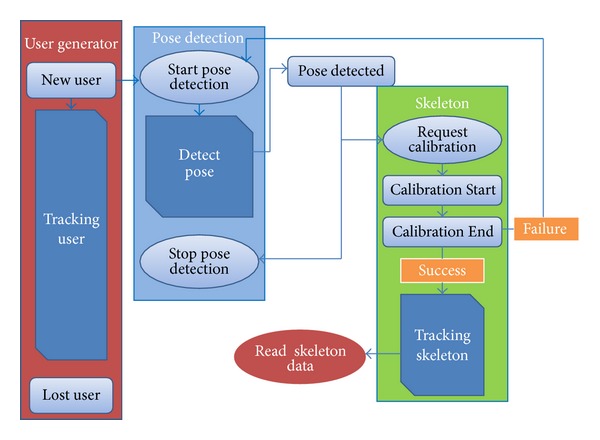
Skeleton detection flowcharts of the Kinect.

The skeleton detection technique is the core module of the somatosensory system. As shown in Figure 5, skeleton of the object was detected by the technique called Light Coding. Pose recognition is achieved by combining the 3D depth map and color information of the object. The dynamic posture correction is also performed till the object is out of the camera's range or program termination.

In the program flowchart, the object detection system is opened firstly. When the object appears on the camera's shooting range, “New User” function is called and the pose detection system starts. “New User” and the “Lost User” functions (events callback function) are called when “new object be detected” and “objects leave the detection range for a while.” “New User” program calls pose detection by “Start Pose Detection” and its own “Pose Detected.” “Pose Detected” check whether the number of object limit is out of bound. Once the limits is reached, it calls “Stop Pose Detection” to stop the detection module. “Pose Detected” also calls the skeleton processing unit named “Request Calibration” for the calibration and analysis of human skeleton. When “Request Calibration” is called, the object's skeleton will be analyzed.

Skeleton processing unit calls “Calibration Start” to start to skeleton correction. When the skeleton calibration is completed, “Calibration End” is called. However, “Calibration End” does not represent a successful identification of the object's skeleton. If it is successful, the next stage is “Start Tracking,” allowing the system to start tracking the skeleton calibration data. If it fails, back to pose detection unit and redetect user gestures. When skeleton calibration and tracking skeleton are successful, users call the joint information function to get the object's joints data. The whole human skeleton is established.

### 3.2. The Gesture Tracking Systems and Applications

**Figure 6:**
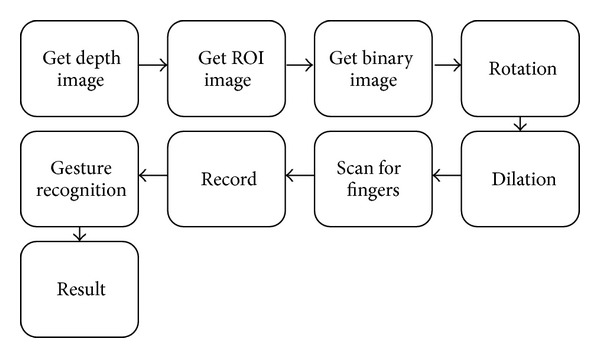
Finger gesture recognition system.

The system expands OPENNI development kit for finger gesture recognition. Users can play puzzle games produced by the system with gestures intuitively. Another application about the gesture recognition system is the Microsoft PowerPoint presentation software. The speaker can switch slides with gestures. Since the Kinect can track the palm's position, we use this information to locate the fingertips' position. With the fingertips' position, finger gesture can be extracted through the number of the fingertips and the angle between the fingertips. Detail system flowcharts can be found in Figure 6.

Each module of the system shown in Figure 6 will be described as follows.

(1)* Get ROI (Region of Interest) Image*. The rough area of the palm is extracted using OPENNI for further analysis and identification. The size of the palm is defined as follows:

(1) $$\begin{array}{c} \text{PlamSize}=\frac{1}{\left(\text{handDepth}\right)\times k_{1}}, \end{array}$$

where the “handDepth” is the palm position depth and *k*_1_ is a constant and is set as 20000 in the experiment. 

(2)* Get Binary Image*. Based on the depth values of each pixel in palm image, the real palm region can be defined as follows:

(2) $$\begin{array}{c} I_{\text{palm}}=\{\begin{array}{cc} 1, & \text{the}\;\text{depth}\;\text{distance}\;\text{of}\;\text{the}\;\text{pixels}\;i\leq d_{\text{plam}},\\ 0, & \text{the}\;\text{depth}\;\text{distance}\;\text{of}\;\text{the}\;\text{pixels}\;i>d_{\text{plam}}. \end{array} \end{array}$$

**Figure 7:**
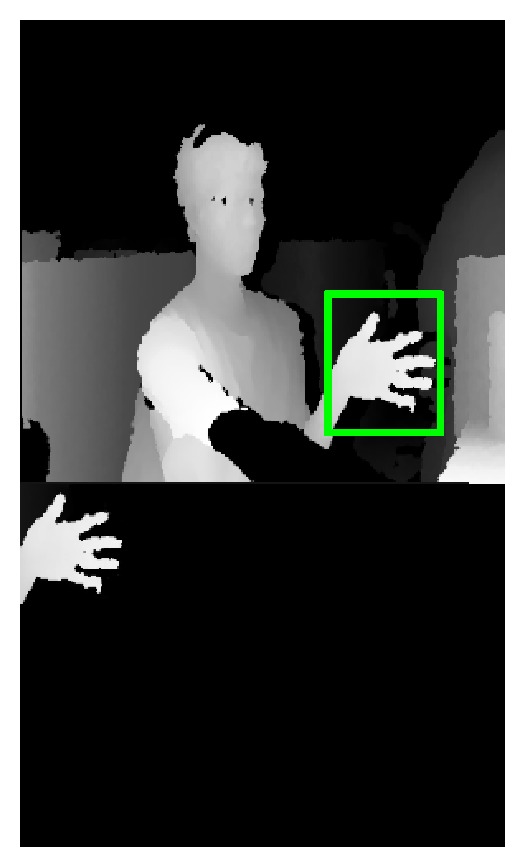
Binary image of the palm.

*d*_plam_ is the depth value of the central pixels of the palm. The upper image of the Figure 7 shows the palm detection result. The bottom image of Figure 7 is the binary image of real palm region. 

(3)* Rotation*. For image normalization, each pixel (*x*, *y*) in the binary image of palm is rotated according to the following:

(3) $$\begin{array}{c} x^{\prime }=x\cos\theta +y\sin\theta ,\\ y^{\prime }=-x\cos\theta +y\sin\theta . \end{array}$$

However, the center axis of the palm needs to be shifted to fit the 2D coordinates using the following:

(4) $$\begin{array}{c} x^{\prime }-h=\left(x-h\right)\cos\theta +\left(y-k\right)\sin\theta ,\\ y^{\prime }-k=-\left(x-h\right)\sin\theta +\left(y-k\right)\cos\theta . \end{array}$$

Given the positions of the palm and wrist, the angle *θ* between the palm orientation and the horizontal direction can be calculated by the following:

(5) $$\begin{array}{c} \theta =\cos^{-1}\left(\frac{a^{2}+c^{2}-b^{2}}{2ac}\right). \end{array}$$

**Figure 8:**
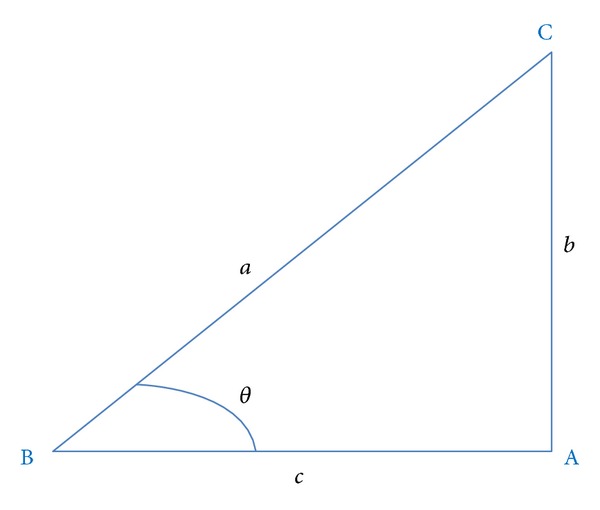
The illustration of the symbols used in (5).

**Figure 9:**
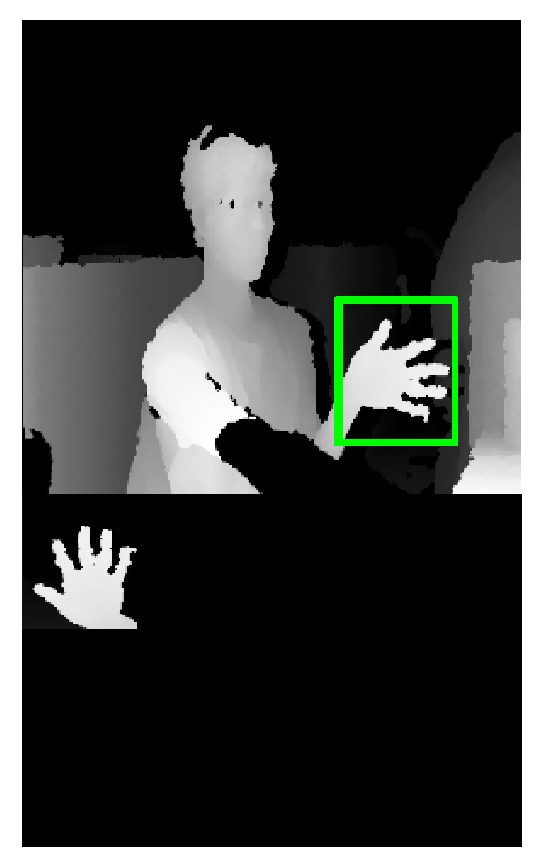
The image at the bottom shows the palm area rotated.

See Figure 8 for an explanation of (5), where the points B and C represent the positions of wrist and palm, respectively. 

(4)* Denoising by Dilation*. To eliminate the noises or the so-called white spots, the morphological dilation operations are employed on the rotated binary image. Note that those noises are generated by the noninteger results calculated by applying (5). There are some white spots on the rotated binary image; see Figure 9 for an illustration. Dilation is a popular morphological operation in the image processing domain and usually used to fill up the small holes spread in a binary image. Simply speaking, the pixel with value 0 (white) in the input image is set to value 1 (black) in the output image if any of its “connected neighbors” are with value 1. Usually, the 8 connected neighbors or 4 connected neighbors are used. In our experiments, the selection of 4 connected ones shows good performance. 

(5)* Finding the Fingertips*. After obtaining the rotated and denoised palm area, we apply Algorithm A below to find the possible positions of all fingertips.

*Algorithm A*

*Step 1*. Scan the binary image from top to bottom to get the first pixel with value 1, and put the pixel in Queue (enqueue operation).

*Step 2*. Retrieve the first pixel from Queue (dequeue operation) and check the values of its 5 neighbors positioned at its right, bottom-right, bottom, bottom-left, and left. Put its neighbor pixels in Queue if the pixels are with value 1 and then set all these pixels to value 0. 

*Step 3*. Repeat Step 2 until the Queue is empty. 

*Step 4*. Repeat Step 1 until the binary image is scanned to its bottom.

**Figure 10:**
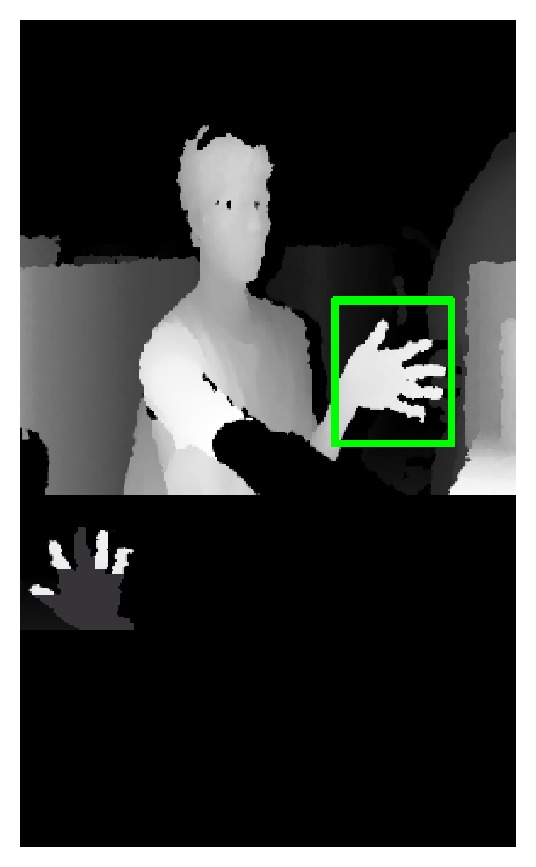
The image at the bottom shows the results after finishing the first round of Step 3.

**Figure 11:**
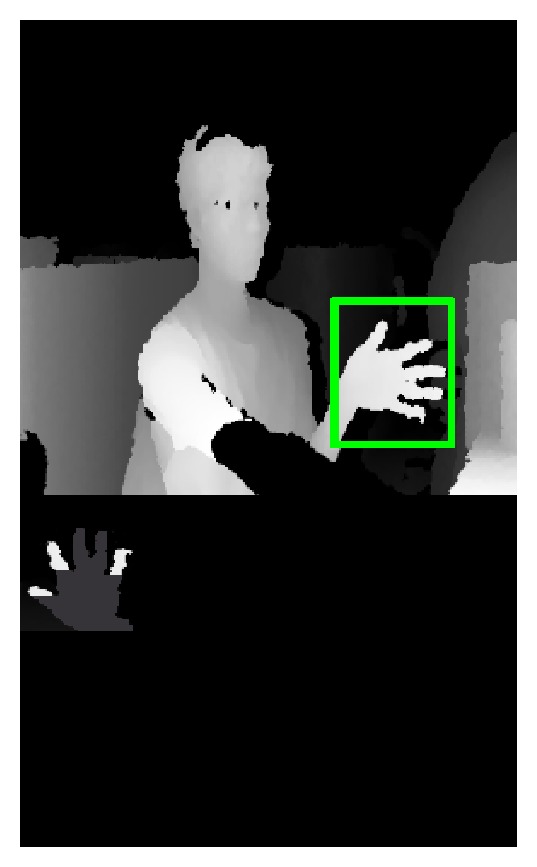
The image at the bottom shows the results after finishing the second round of Step 3.

To understand the algorithm above more clear, let us see the example shown in Figure 10. The bottom image in Figure 10 shows the results after finishing the first round of Step 3 and in this case the middle finger is detected. The grey color denotes the area eliminated in Step 2. The bottom image in Figure 11 shows the results after finishing the second round of Step 3 and in this case the ring finger is detected.

**Figure 12:**
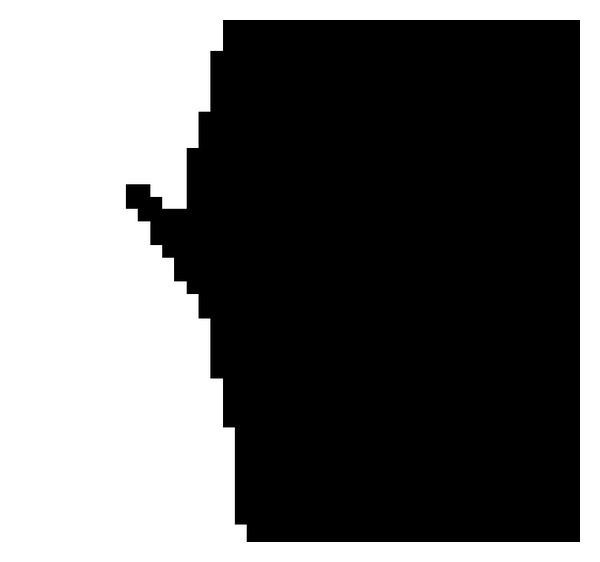
The protrusion at the boundary of one finger is erroneously detected as another finger.

Note that the time complexity of Algorithm A is *O*(*n* ^2^), where *n* is the number of pixels. 

(6)* Recording the Positions of Real Fingertips*. In ideal case, there are at most 5 fingertips detected. But the erroneous protrusions due to noises could be detected as fingertips; see Figure 12 for such a possible situation.

After getting the candidates of fingertips and in order to eliminate the erroneous fingertips and record the real ones, Algorithm B below is then applied.

*Algorithm B*

**Figure 13:**
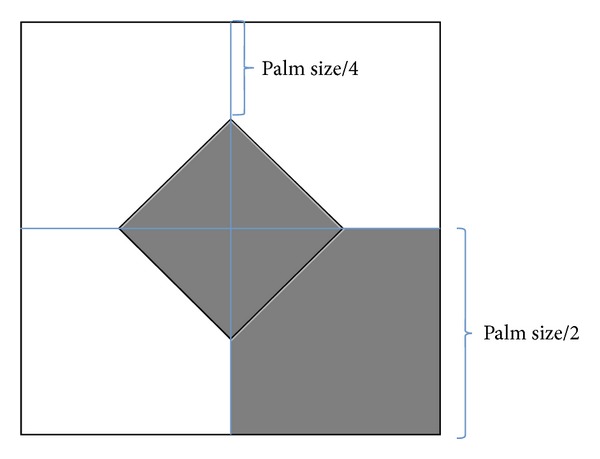
The grey color indicates the “Nonfingertip appearing area,” and this area is derived from the right hand.

*Step 1*. Eliminate those candidates residing on the “Nonfingertip appearing area” indicated by the area with grey color in Figure 13. 

*Step 2*. Cluster the candidates fingertips detected in previous module according to their proximity calculated by city block distance. 

*Step 3*. The top 5 (at most) candidates in the image, one from each clustering group, are selected as the real fingertips. 

(7)* Gesture Recognition*. The two positions of palm and wrist had been obtained through the OPENNI framework in advance and, with these two positions at hand, we can detect the positions of fingertips using the modules described previously. Therefore, the gesture can be recognized by the positions, distances, and angles between these positions. In this study, the clench gesture denotes that the user wants to grasp the object appearing at the corresponding position of the hand. And moving the hand with clench gesture indicates that the user wants to move the 2D object or rotate the 3D object. The opening gesture will break the link between the hand and its corresponding object. In addition, many other gestures can also be recognized and thus different applications can be created.

### 3.3. The Construction of Somatosensory Game

Based on the teaching theory and advantages illustrated by the scholars above, the somatosensory games proposed in this study integrate five elements: challenge, interaction, rules, goal, and social relationship. We propose two somatosensory games: puzzle game and geometric game.

**Figure 14:**
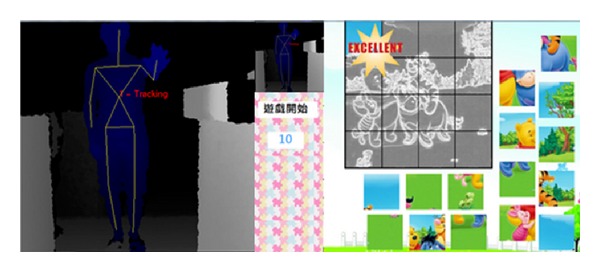
The procedure of puzzle game.

**Figure 15:**
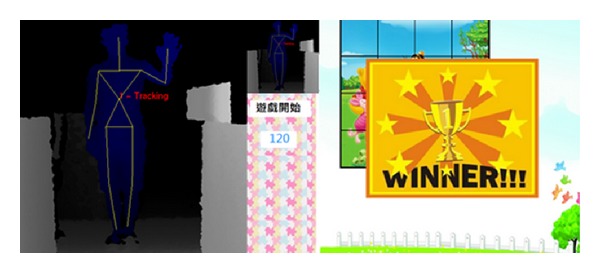
The completeness of puzzle game.

In the puzzle game, the first phase is scoring model, students can freely explore the fun of somatosensory system and fulfill the puzzle mission. Each operation will bring back feedbacks; for example, the action of capturing images would produce corresponding sound and icons to remind students, and puzzles placed into the correct position and the wrong position will trigger a corresponding animation and sound alarm, respectively, to reward and punish students. The second phase is time keeping model; after the first phase, the students have been familiar with the operation of the game. In this phase, the students will be put into the time pressure, to improve and train their concentration. Under this time keeping model, as time goes by, warning animation will become increasingly apparent and sound will be more rapid to force the students under time pressure with high concentration completing the puzzle as Figures 14 and 15 show. By this way, the concentration and logic of the students can be effectively improved.

In the first phase of scoring model, the main purpose is guiding the users to be familiar with the operation of the somatosensory system and raise their interests. In this stage, the elements of intrinsic motivations such as interaction, rules, and goal are immersed into the users. Then, in the second stage, the main goal is to raise the attention of users to the puzzle game and their reasoning ability. In this second stage, the elements of intrinsic motivations such as challenge, risk, and goal are immersed into the users.

**Figure 16:**
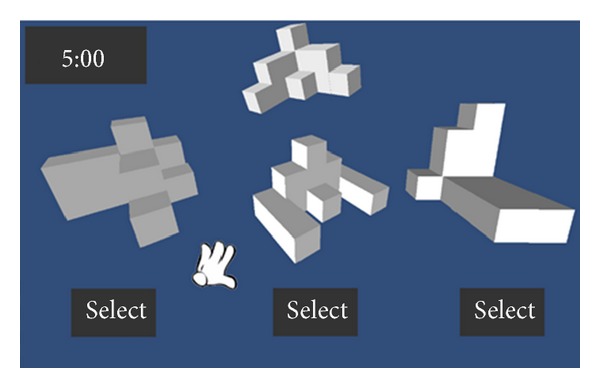
The illustration of geometric game.

**Figure 17:**
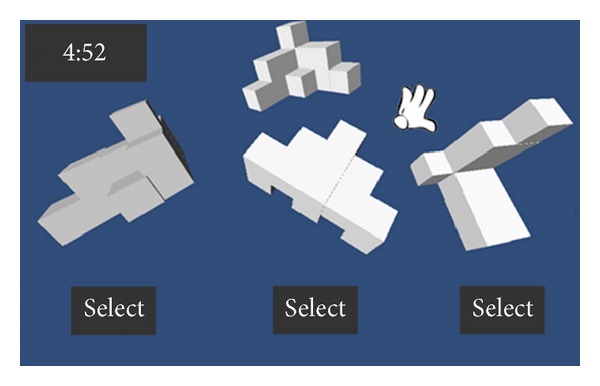
Another illustration of geometric game.

Next, we also proposed another geometric game and the main objective of the game is to use a virtual 3D objects with the somatosensory system that allows students to intuitively and easily view and rotate the virtual 3D objects and learn more about 3D geometric concepts through this way. The game is divided into two stages. The first stage is paper testing, the learners should be complete 25 questions within a fixed time. Each question consists of four geometric patterns, in which a geometric pattern is the target and the rest are the optional ones. Learners choose from three optional ones having the same volume with the target. If the answer is correct, they gain the bonus points. At this stage, the students evaluate their ability of geometry reasoning. Then, the second phase of gameplay consists of 25 questions. Each question would reveal a target object at the top of the screen, and there are three different candidate objects at the bottom. The user can optionally view and rotate these objects and select one of three objects within the stipulated time. As Figures 16 and 17 illustrate, if the selected objects have the same volume with the target object, it is correct otherwise an error.

### 3.4. The Application of Somatosensory in Learning

With the growing technical of somatosensory system, it has become common in the entertainment game industry. But somatosensory can not only bring people the joy of playing games. Unlike the usual way, it is a better and more appealing man-machine interactive media and also an excellent learning tool in the field of digital learning (especially young children learning). There are already many scholars and industry investing in developing. The application of somatosensory is wide; in addition to the use of games, medical industry, education institutions, and police investigation could take advantage of this unique and fun way to get a different feeling in the learning application. When somatosensory is applied in learning, students' interest in learning, intuitive in operation would be enhanced via manipulating the virtual characters and objects. In addition, similar to the authenticity of such a learning a learning environment; students will have a simple and intuitive feeling to increase the interest in study. For example, the learning of dancing in general environment must only be to imitate the way through movies. With the help of the somatosensory system, you can create a 3D virtual environment in the meanwhile. Then the learning performance could be estimated via student's dace choreography. Mathematical learning can also be used to aid in the somatosensory system. For example, facing the geometry problems with space concept, you can manipulate 3D models through somatosensory designed for students to experience it. Students can not only gain a more intuitive and more intense sensory stimulation and logic training, but also can enhance students' interest in learning. In this study, the main consideration is intuitive, interactive, and fun. It combines the original puzzle and somatosensory, to attempt to allow students to intuitively operate the puzzle in virtual world and to train the logical thinking of students through puzzles and enhance the students' interest in learning to achieve happy learning.

### 3.5. Analysis of Learning Speed and Experience

This study divided puzzle game into two phases: scoring mode and timing mode. In score mode, the main consideration is for students to familiarize with the somatosensory environment and stimulation. It encourages students to try to complete the puzzle with scoring points. The second phase timing mode is to analyze through students' learning speed and score. The effects of somatosensory on learning speed could be analyzed by estimating students' scoring points under the pressure of time. On the other hand, geometric learning games are also divided into two stages. The first stage is in the traditional way on paper test; the second stage is to allow students to manipulate virtual objects. After completion of the test, we survey the feelings and perception of somatosensory games as the basis for analysis of learners.

## 4. Experimental Results

**Figure 18:**
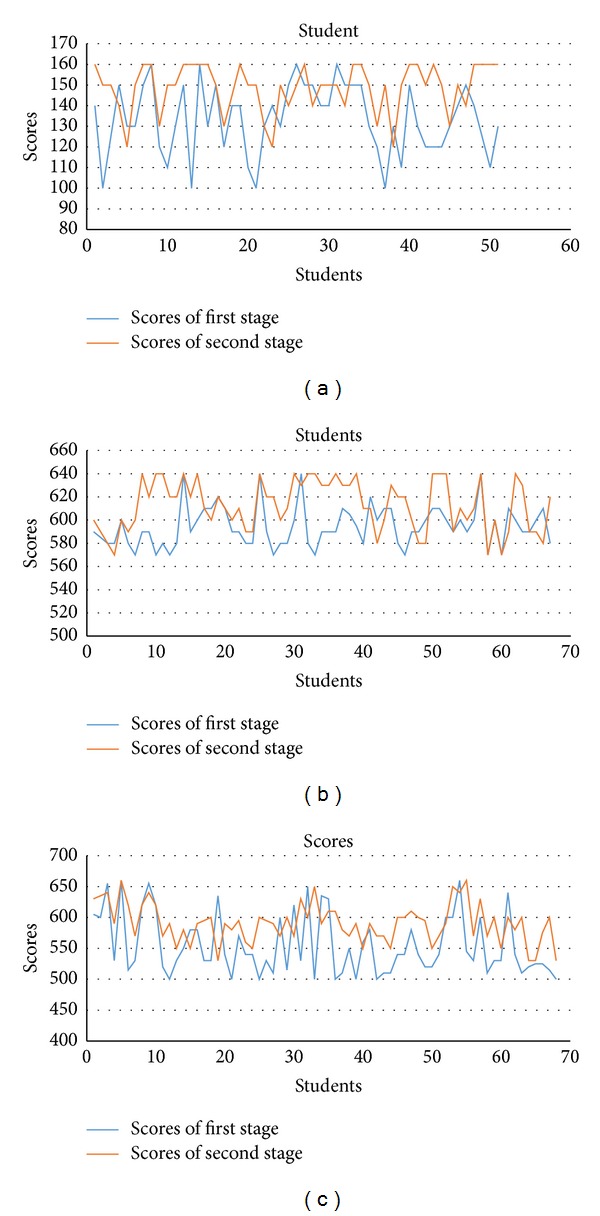
(a) is the result of G1 students. (b) is the result of G3 students. (c) is the result of G5 students.

In this section, experimental results conducted on the puzzle game and geometric game are illustrated to demonstrate the effectiveness of the proposed method. In the puzzle game, there are two modes in this experiment. Each mode processes one game teaching. In puzzle game, the first mode adopts scoring mode game, whose point is to make learners somatosensory game and be familiar with the operation of puzzle environment. The second mode adopts timing mode, whose purpose is to measure and observe the learners' learning ability and their attention and logical thinking on puzzle game under time pressure. There will be two times of timing mode. We record separately the learner's scores as reference of learning speed. The practical environment is a multimedia computer classroom. Besides a projector, there are Kinect camera and 25 computers. The teacher's operation teaching time is one class per unit. Six minutes in class for each learning comprehension test and 50 minutes for each class. Hence, two units consume two classes. The place of this experiment is in the multimedia computer class. Two learners use one computer. The researcher of this experiment is not the class teacher. The teacher teaches with the materials. Before teaching, the researchers explain the purpose of the research and remind the teachers' materials and the operation and notes of this practice. During the game, the researchers will aid the teachers on teaching and assist the learners to solve the problems of learning. The learners of this research are 51 G1 students, 27 boys and 24 girls; 67 G3 students, 38 boys and 29 girls; 68 G5 students, 38 boys and 30 girls. There are total 186 students. The experiment is divided into 3 levels, easy, medium, and difficult. The difficulty is based on the numbers of puzzles: 16, 64, and 81 puzzles. There are two parts of results. Part 1 is to analyze the learners' learning speed and their scores. See Figure 18.

According to Figure 18, we can find that the second test scores are mostly higher than the first test scores. This is because after the first test, the learners are getting familiar with the somatosensory game. Therefore, they get better scores on the second test. Because each learner's score on these two stages is not the same, it presents the difference between learners' learning speed on somatosensory system. We can also understand the effect and influence of learners on somatosensory system with the survey on second stages. The contents of the survey are as Table 3(a). The results of survey are as Table 1. As shown in Figure 18, the second test scores are mostly higher than the first ones. The learners are familiar with the somatosensory game after the first test. Therefore, they get better scores on the second test. According to different learner's score on these two tests, the analysis of learning speed with the somatosensory system can be obtained. To get the effect and influence of learners on somatosensory system, we provide the questionnaire as shown in Table 3 to analyze the impact and feelings of learner with the somatosensory system. The results are listed in Table 1. From the result, the new stimulus brought by somatosensory system can intrigue learners' interest on this game, which makes learners concentrate on the game and develop their logical and thinking ability into the game. They also meet the challenge and gain achievement among it.

**Figure 19:**
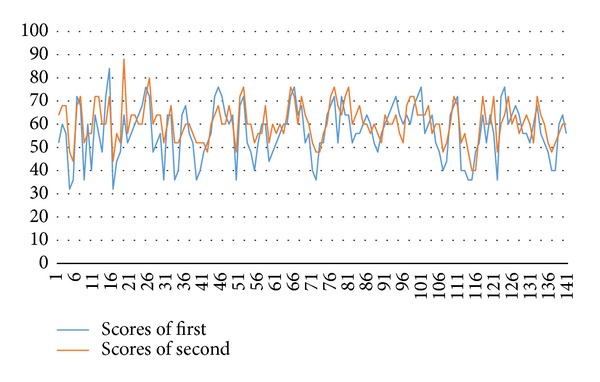
Distribution of test scores.

In addition, in the geometry game, there are two steps in this experiment. We use traditional paper test in the first step. The content of test sets an object as a goal which is the same size with targeted object and there are 25 questions with 3 choices. Five points for each question. The questions in the second stage are the same with those in the first stage. However, they are presented with 3D virtual objects and the learners operate it with somatosensory system as an interface, which is based on hand gestures to allow learners to rotate and see the 3D virtual objects. This research is practiced in the same place with the puzzle game. The subjects are 141 G5 students, 72 boys and 69 girls. The aim is to observe the level of how learners enjoy and how much interest is intrigued, the learning speed, and learning status when they face new learning approaches. This part is conducted by a survey. The result of test is as Figure 19. The result of survey is as Table 2. The content of survey is as Table 3(b).

As we can see in Figure 19, learners score higher with new test approach than traditional paper test. Especially the group of lower scores, they progress enough. This is because learners can rotate the virtual objects through somatosensory system. By this kind of interactive approach, the freedom of learning has been improved and the learners understand geometry better. From the result of the survey, most learners think that this new approach is really helpful.

## 5. Conclusions

Somatosensory system is an interactive media system which has risen recently. Because of its directness, the users gain more feedback during the operation. Therefore, in this study, we proposed a somatosensory based learning system and discuss the influence and the change brought by somatosensory system. In somatosensory system, we create a puzzle game suitable for students, which is built by body skeleton and human behavior. Also, by doing this, we observe how helpful somatosensory game is and if the learners can learn happily during this somatosensory game. From the result, most learners hold a positive attitude when using this new approach to learn. Among all the factors, including interest/enjoyment, perceived competence, effort/importance, pressure/tension, enjoyment factor is the learners gain the best. Because the specialty and fun of somatosensory game intrigues learners' interest, they learn and play simultaneously. This kind of somatosensory system provides users with different ways to interact with computers and draw more attention from users. Meanwhile, the users can operate it more fluently and interactively.

## Figures and Tables

**Table 1:** Results of the survey.

Learning state	Mean (M)	Standard deviation (SD)	Sample (*N*)
Interest/enjoyment	5.47	1.62	186
Perceived competence	4.69	1.81	186
Effort/importance	5.24	1.44	186
Pressure/tension	2.74	2.00	186

**Table 2:** Results of the survey.

	Score of average (M)	Standard deviation (SD)	Sample (*N*)
Great help in learning	5.68	1.64	141
Little help in learning	3.94	1.49	141
No help for learning	3.24	1.93	141

**Table:** (a)

After you finished the game, please fill up the form below. Make scores of items according to what you felt in the game.							
(1) I felt very happy when I was playing the game	7	6	5	4	3	2	1
(2) I think I had done well in the game	7	6	5	4	3	2	1
(3) I did a lot of effort in the game	7	6	5	4	3	2	1
(4) I got no pressure when I was playing the game	7	6	5	4	3	2	1
(5) This game is very interesting	7	6	5	4	3	2	1
(6) I think I can do a better job next time	7	6	5	4	3	2	1
(7) I had tried to perform better in the game	7	6	5	4	3	2	1
(8) I felt nervous when I was playing the game	7	6	5	4	3	2	1
(9) I think this game makes me feel happy	7	6	5	4	3	2	1
(10) I'm satisfied to my performance in the game	7	6	5	4	3	2	1
(11) I really do a lot of efforts in the game	7	6	5	4	3	2	1
(12) I felt relaxed when I was playing the game	7	6	5	4	3	2	1
(13) Playing the puzzle game makes me very attentive	7	6	5	4	3	2	1
(14) I think I'm good at playing the puzzle game	7	6	5	4	3	2	1
(15) I think this activity is important to me	7	6	5	4	3	2	1
(16) I feel anxiety when I was playing the game	7	6	5	4	3	2	1
(17) I think playing the game makes me feel happy	7	6	5	4	3	2	1
(18) I think I'm good at playing puzzle games	7	6	5	4	3	2	1
(19) I spend a lot of energy in this game	7	6	5	4	3	2	1
(20) This activity makes me feel pressure	7	6	5	4	3	2	1

**Table:** (b)

After you finished the game, please fill up the form below. Make scores of items according to what you felt in the game.							
(1) This game is helpful for learning geometric concepts	7	6	5	4	3	2	1
(2) This game is not very helpful for my learning	7	6	5	4	3	2	1
(3) I think I learned nothing in this activity	7	6	5	4	3	2	1
(4) I was very focused on playing the game	7	6	5	4	3	2	1
(5) I think this game is not very fun	7	6	5	4	3	2	1
(6) I felt nervous when I was playing the game	7	6	5	4	3	2	1
(7) I have learned a lot from this activity	7	6	5	4	3	2	1
(8) I think the learning effect from this activity is limited	7	6	5	4	3	2	1
(9) I learned nothing from this activity	7	6	5	4	3	2	1
(10) I'm satisfied to my performance in the game	7	6	5	4	3	2	1
(11) This activity has no effect on me	7	6	5	4	3	2	1
(12) I think this activity is not good	7	6	5	4	3	2	1
(13) I think learning by playing game is a good way	7	6	5	4	3	2	1
(14) I think learning by playing game is a common way	7	6	5	4	3	2	1
(15) I think learning by playing game is not good	7	6	5	4	3	2	1
(16) I think this way to learn is better than traditional way	7	6	5	4	3	2	1
(17) I think this learning way is not special for me	7	6	5	4	3	2	1
(18) I prefer traditional way to learning	7	6	5	4	3	2	1
(19) I spend a lot of energy in this game	7	6	5	4	3	2	1
(20) I'm not interested in this activity	7	6	5	4	3	2	1
(21) This activity makes me feel pressure	7	6	5	4	3	2	1
